# Noninvasive Physiologic Assessment of Coronary Stenoses Using Cardiac CT

**DOI:** 10.1155/2015/435737

**Published:** 2015-01-20

**Authors:** Lei Xu, Zhonghua Sun, Zhanming Fan

**Affiliations:** ^1^Department of Radiology, Beijing Anzhen Hospital, Capital Medical University, Beijing 100029, China; ^2^Discipline of Medical Imaging, Department of Imaging and Applied Physics, Curtin University, Perth, WA 6845, Australia

## Abstract

Coronary CT angiography (CCTA) has become an important noninvasive imaging modality in the diagnosis of coronary artery disease (CAD). CCTA enables accurate evaluation of coronary artery stenosis. However, CCTA provides limited information on the physiological significance of stenotic lesions. A noninvasive “one-stop-shop” diagnostic test that can provide both anatomical significance and functional significance of stenotic lesions would be beneficial in the diagnosis and management of CAD. Recently, with the introduction of novel techniques, such as myocardial CT perfusion, CT-derived fractional flow reserve (FFR_CT_), and transluminal attenuation gradient (TAG), CCTA has emerged as a noninvasive method for the assessment of both anatomy of coronary lesions and its physiological consequences during a single study. This review provides an overview of the current status of new CT techniques for the physiologic assessments of CAD.

## 1. Introduction

Coronary CT angiography (CCTA) has been widely used as an effective noninvasive imaging modality in the diagnosis of coronary artery disease (CAD) [1]. Multiple-center studies have shown that CCTA allows reliable detection of coronary artery stenoses with high sensitivity and specificity as compared to conventional coronary angiography [2, 3]. Due to the remarkably high negative predictive value and noninvasive nature, the main strength of CCTA is its excellent ability to exclude significant CAD in selected patients with intermediate pretest probability [1, 4, 5]. Therefore, CCTA is now well established as an effective “gatekeeper” to invasive coronary angiography and may reduce the rate of normal coronary angiography and improve cost efficiency [6]. Noninvasive assessment of coronary plaque is important for coronary risk stratification. In addition to accurate evaluation of luminal narrowing, CCTA is now a promising approach for noninvasive detection of coronary plaque characteristics, including plaque composition and morphology, and it may therefore contribute to coronary risk stratification [7–9]. A previous study has shown that CCTA-based plaque characterization provides valuable information for the prediction of major cardiovascular events [10]. CCTA not only demonstrates high diagnostic accuracy, but also shows high prognostic value in CAD with very low rate of adverse cardiac events occurring in patients with normal CCTA finding and significantly high rate of these events in patients with obstructive CAD [11–13]. CCTA is now a mature diagnostic imaging modality that can provide an effective means to safely guide clinical decision making [14]. One of the limitations of CCTA is that the presence of extensively calcified plaque may cause overestimation of the degree of coronary stenosis, thus resulting in low positive predictive value and potentially leading to increased downstream testing [15–17].

Detection of the physiological severity of intermediate coronary lesions has significant implications for the diagnosis, prognosis, and optimal treatment [18, 19]. It has been shown that only patients with a hemodynamically significant coronary stenosis benefit from revascularization regardless of the severity of underlying stenosis [20, 21]. However, in its current form, CCTA is limited in the physiological assessment of coronary atherosclerosis and, therefore, it cannot efficiently discriminate hemodynamically from non-hemodynamically significant stenosis [1]. Studies have shown that a 50% stenosis identified by CCTA is a poor predictor of ischemia [22–24]. Hence, on identification of the significance of CCTA findings, patients often require additional functional tests, as the physiological significance of many lesions remains uncertain. Given these considerations, a noninvasive “one-stop-shop” diagnostic test that can provide both anatomical and hemodynamic significance of stenotic lesions would be beneficial in the diagnosis of CAD. Recently, with the introduction of novel techniques, such as myocardial CT perfusion, noninvasive FFR_CT_, and transluminal attenuation gradient, CCTA has emerged as a noninvasive method for the assessment of both anatomy of coronary lesions and its physiological consequences during a single study. This review provides an overview of the current status of new CT techniques for the physiologic assessments of CAD.

## 2. Myocardial CT Perfusion Imaging

Nuclear medicine for a long time has played an important role in the noninvasive evaluation of known or suspected coronary artery disease. Over the past decades, myocardial perfusion imaging (MPI) using single photon emission computed tomography (SPECT) or positron emission tomography (PET) has been well established as the reference standard for the diagnosis and decision making in patients with known coronary artery disease [25, 26]. A meta-analysis demonstrated that the pooled sensitivity and specificity of SPECT-MPI was 77% and 77% on a patient level for the evaluation of functional ischemia with fractional flow reserve as the reference standard [27]. However, SPECT is still limited by low spatial resolution and may miss small or subendocardial areas of hypoperfusion. Attenuation artifact has long been recognized as a major factor limiting the specificity of SPECT for the detection of myocardial perfusion defects [28]. Furthermore, SPECT-MPI may miss some patients with balanced three-vessel disease, as the technique relies on identifying relative differences in perfusion between adjacent myocardial territories [29]. Compared with SPECT-MPI, PET-MPI provides higher spatial resolution, accurate attenuation correction, and lower radiation doses for the detection of coronary artery disease [30, 31]. PET perfusion imaging has an overall sensitivity of 92% and a specificity of 85% for the detection of coronary artery disease [32]. However, because of cost and logistics, this approach is not widely available and is not accepted as a clinical routine. The use of hybrid imaging techniques, such as SPECT-CT or PET-CT has the advantages of both functional and anatomic imaging. However, these modalities still require two separate imaging procedures, resulting in additional cost and radiation exposure [22, 30]. The advantage of cardiac magnetic resonance imaging (CMR) over myocardial SPECT is high spatial resolution and not using ionizing radiation [33]. A meta-analysis of CMR perfusion study revealed a sensitivity of 91% and specificity of 81% for the diagnosis of CAD on a per-patient level [34]. However, CMR examination is time-consuming as it has long procedure times and limited accuracy in quantification. MR coronary angiography has not been widely accepted as a practical diagnostic tool for CAD. Technical advances with improved spatial and temporal resolution and subsequently reduced scan time and radiation exposure have allowed cardiac CT to simultaneously assess both coronary anatomy and myocardial perfusion.

## 3. First-Pass Myocardial CT Perfusion

Myocardial CT perfusion imaging (CTP) can be performed in two ways: first-pass static perfusion imaging and dynamic CTP. First-pass CCTA imaging has the potential to assess the physiologic significance of coronary artery lesions [35]. In this strategy, rest myocardial CTP is obtained from the images usually acquired during a CCTA examination performed in rest condition. And stress CTP is acquired by an additional CCTA study performed under myocardial stress [36, 37].

By using resting CCTA images, coronary artery stenosis and myocardial perfusion may be evaluated simultaneously using the same raw data without the need for further scans, or additional radiation and contrast. However, the value of rest myocardial CTP for the evaluation of myocardial ischemia is uncertain. There have been some reports investigating the detection of myocardial ischemia at rest using multidetector CT (MDCT). Iwasaki and Matsumoto [36] studied the incidence of myocardial perfusion defect by 64-MDCT at rest in patients with significant stenosis and the effect of coronary revascularization therapy on myocardial perfusion. The results demonstrated that a significant percentage of patients with significant coronary stenosis showed myocardial perfusion defect by 64-MDCT at rest, and most of these perfusion defects improved after revascularization therapy. The results indicate that CCTA has the potential for the detection of myocardial ischemia at rest. A study by Busch et al. [38] showed that, in comparison to combined SPECT and CCTA, myocardial CTP using resting CCTA images identifies myocardial infarction with high sensitivity (90%), good specificity (80%), and high negative predictive value (NPV) (94%). The presence of hypoperfusion on CTP suggests either myocardial infarction or ischemia with high predictive power (92%). In contrast with prior studies, Troupis et al. [39] assessed the sensitivity of 320-detector CT for the detection of myocardial density changes based on rest CCTA by comparing patients with severe coronary artery stenoses (>75%) and patients with no stenosis. Comparison of ischemic myocardial segments with nonischemic segments demonstrated no significant difference in myocardial density, confirming that myocardial ischemia cannot be reliably detected on rest CCTA. Rest myocardial CTP may have a role in the assessment for myocardial infarction [40, 41]. However, performance of only rest myocardial CTP may be insufficient to reliably rule out reversible ischemia [42].

Stress CTP is performed under pharmacological administration of stress agents, such as adenosine, dipyridamole, or regadenoson, similar to nuclear medicine MPI [43]. In the ischemic cascade, stress perfusion abnormalities are more sensitive than wall motion abnormalities [44]. Myocardial CTP protocols typically include a stress and a rest phase acquisition similar to a nuclear myocardial perfusion imaging examination. When the time interval between the 2 scans is short, the myocardium in the second study may be contaminated by previous injection of contrast material which may decrease the sensitivity for detection of ischemic myocardium. Delay time of 10–20 minutes should be used between the two CT scans to allow wash out of contrast from the myocardium. During rest phase acquisition, prospectively ECG-gated imaging can be performed with tube current and voltage tailoring to body mass index to reduce radiation dose. Stress phase imaging is performed with pharmacologically induced stress. The most commonly used stress agent in perfusion imaging is vasodilator agent adenosine. Adenosine is continuously infused at a dose of 0.14 mg/kg per minute over 3–5 minutes. Stress scan acquisition was performed at peak contrast enhancement with a second bolus injection of contrast [37]. Hypoattenuation areas in the myocardium on CTP represent ischemic myocardium [45]. Generally, if hypoattenuation areas are visualized at stress imaging only, this may indicate reversible myocardial ischemia; if hypoattenuation is visible at rest, this is suggestive of myocardial infarction [43].

Studies have been conducted to investigate the diagnostic performance of stress CTP for the evaluation of CAD. Blankstein et al. [46] studied the diagnostic accuracy of adenosine-stress CTP for identification of hemodynamically significant stenosis compared with nuclear MPI using invasive angiography as the reference standard. The stress CTP had 96% sensitivity, 73% specificity, and 98% NPV on per-vessel basis for the detection of stenosis ≥70%. Adenosine stress CTP has comparable diagnostic accuracy to SPECT in detecting stress-induced myocardial perfusion defects. The comparison of stress myocardial perfusion imaging with invasive fractional flow reserve (FFR) has been performed by Ko et al. [47]. In this study, forty-two patients with significant stenosis on invasive angiography were included with use of 320-detector row CT. The sensitivity, specificity, positive predictive value (PPV), and NPV of CTP on per-vessel territory were 76%, 84%, 82%, and 79%, respectively. Combining a ≥50% stenosis on CCTA and perfusion defect on CTP was 98% specific for ischemia, while the presence of normal perfusion on CTP and <50% stenosis on CCTA was 100% specific for exclusion of ischemia. Feuchtner et al. [33] reported stress myocardial CTP using dual-source CT (DSCT) high-pitch mode for detecting reversible ischemia with the comparison of MRI. Stress CTP had sensitivity of 96%, specificity of 88%, PPV of 93%, and NPV of 94% on per-vessel basis. The accuracy increased from 84% to 95% after adding stress CTP to CCTA. The results suggest that stress myocardial CTP imaging with 128-slice high-pitch DSCT is feasible for accurate detection of reversible myocardial ischemia.

CORE320 is a multicenter multinational diagnostic study which was designed to evaluate the diagnostic performance of 320-MDCT for detecting coronary stenosis and myocardial perfusion deficits in patients with suspected CAD compared with conventional coronary angiography and SPECT MPI [48]. In this study, 381 patients who underwent combined CCTA-CTP and SPECT-MPI prior to conventional coronary angiography were enrolled from sixteen centers [49]. With the advantage of temporal uniformity achieved by the use of wide detector 320-MDCT system, the diagnostic accuracy of combined CCTA and CTP for detecting or excluding flow-limiting CAD was 0.87 on a patient-basis (defined by area under the receiver operator characteristics curve, ROC). The sensitivity, specificity, PPV, and NPV of integrated CCTA stenosis and CTP perfusion deficit were 80%, 74%, 65%, and 86%, respectively. The accuracy of CCTA was significantly improved by adding CTP at both the patient and vessel levels. The study indicates that the combination of CCTA and CTP correctly identifies patients with flow-limiting CAD. More recently, one study showed that combining CCTA and CTP improves the diagnostic performance of coronary in-stent restenosis and CAD in patients with stents compared with CCTA alone [50]. The study is meaningful for clinical decision making in these symptomatic patients.

The use of adenosine during stress CTP may increase the heart rate. CTP is prone to motion artifacts particularly in patients with high heart rate, which may compromise the evaluation of myocardial perfusion. Combined CCTA/CTP has been proven to have better diagnostic performance than CCTA alone. Clinical outcome studies are still needed to determine the effectiveness of stress CTP in evaluating patients with known or suspected CAD compared with other modalities.

## 4. Dual-Energy Myocardial CT Perfusion

**Figure 1:**
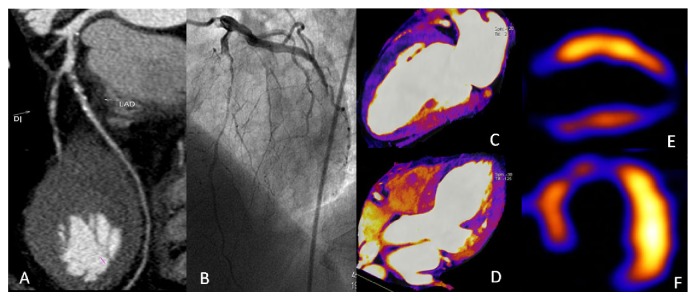
Curved multiplanar reformation of CCTA (A) shows severe stenosis in the left anterior descending artery (LAD). Coronary angiography (B) demonstrates the occlusion in the proximal LAD. DE-CTP images show hypoperfusion at the anterior, apical, septal, and inferior walls in the two and four-chamber views (C and D). SPECT-MPI images reveal perfusion defects in the same regions (E and F).

Recently, CTP has been performed by using the dual-energy mode. Dual-energy CT (DECT) is based on the principle that body tissues and iodinated contrast have specific spectral characteristics at different energy of X-rays [51, 52]. DECT acquisition can be classified into two categories: source-oriented and detector-oriented. Currently, two popular approaches for performing DECT are either with dual-source system to produce X-rays with two different tube voltages or with a single X-ray tube switching rapidly between low and high kilovoltage during a single scan [53]. After acquisition of the high- and low-energy data, the iodine content within the myocardium can be determined using the dedicated cardiac DECT postprocessing algorithm based on the unique X-ray absorption characteristics of iodine at different kV settings. The color-coded iodine distribution maps superimposed on gray-scale multiplanar reformats of the myocardium in short- and long-axis views are then generated, which can be used for the detection of myocardial ischemia [54] (Figure 1). The DE-CTP protocol commonly involves rest scan and stress testing. The rest acquisition is also used for coronary angiography evaluation. Stress testing under the administration of adenosine is subsequently performed with second bolus of iodine contrast medium [52]. The potential advantage of DECT is that myocardial perfusion defects are more conspicuous on iodine color maps compared with single-energy CT evaluation [52, 55]. Perfusion defects could be visualized by using rest-only DE-CTP [42]. Wang et al. [56] studied 34 patients with abnormal SPECT findings or known CAD using only rest DECT. Compared with SPECT, DECT had 68% sensitivity and 93% specificity for the detection of myocardial perfusion defect. Combining CCTA and rest DECT, the diagnostic accuracy was slightly improved from 86% to 88% when compared with CCTA alone using invasive coronary angiography as the reference standard. The study indicates that combination of DE-CTP and DE-CCTA may improve diagnostic performance compared to CCTA alone for significant stenosis. One study compared stress and rest DECT for the detection of myocardial perfusion defects, and the results indicate that stress DECT has superior performance for the detection of perfusion defects compared with rest DECT with CMR as the reference standard [57]. Ko et al. [58] investigated the incremental value of combined CCTA and DE-CTP for the detection of significant coronary stenoses. The study was performed by adenosine-induced stress DE-CTP and conventional coronary angiography. The sensitivity, specificity, PPV, and NPV of the CCTA alone on a per-vessel basis were 91.8%, 67.7%, 73.6%, and 87.5%, respectively, and these values were 93.2%, 85.5%, 88.3%, and 91.4% for combining CCTA with CTP, respectively. The area under the ROC increased from 0.798 to 0.893 (*P* = 0.004). The results indicate that combined CCTA and CTP may provide incremental diagnostic value compared with CCTA alone for the detection of significant coronary stenoses in patients with CAD, especially in patients with heavily calcified plaques or implanted stents. To evaluate the feasibility and diagnostic accuracy of stress DECT for detecting hemodynamically significant stenosis causing reversible myocardial perfusion defect, the same group studied 41 patients with known CAD compared with stress MR perfusion and conventional coronary angiography. Stress DECT had sensitivity, specificity, and accuracy of 89%, 78%, and 82%, respectively, for detecting segments with reversible perfusion defects compared with MR perfusion. For the detection of vascular territories with reversible perfusion defect that had hemodynamically relevant CAD, stress DECT had 89% sensitivity, 76% specificity, and 83% accuracy compared with conventional coronary angiography. The results demonstrate that stress DECT has the potential to identify stress-induced myocardial perfusion defect in patients with CAD. It should be noted that DE-CTP is more susceptible to motion artifacts during adenosine infusion and beam-hardening artifacts which mimics perfusion defects and led to false positives [59]. Recent studies also suggest that rapid kV-switching projection-based DECT could be an effective technique for eliminating the beam-hardening effect due to the ability to reconstruct monochromatic CT images; thus, it may permit improved quantitative myocardial CTP [60, 61]. Although the limited study results demonstrate that DECT-based evaluation of myocardial perfusion defects has the potential for the assessment of hemodynamically significant coronary stenosis in patients with CAD, more future studies will be required before DECT can be incorporated into routine clinical application.

## 5. Dynamic Myocardial CT Perfusion

Single-phase first-pass CTP is highly dependent on accurate bolus timing and thus the peak attenuation may be missed because of the acquisition of only one sample of the data. A dynamic CT perfusion scan can be performed while the scanning table is stationary or in shuttle mode [62, 63]. During the shuttle mode imaging, data are acquired over a 30-second interval with the table moving forward and backward between both positions to cover a scan range of 73 mm. Dynamic CTP has the advantage of capturing an entire dynamic series of contrast-enhanced myocardium at stress and at rest. From the acquired multiphase CT images, time-density curve (TDC) of the myocardium and its supply arteries are measured. The myocardial blood flow (MBF) and myocardial blood volume (MBV) can be derived from TDC using various mathematical models, leading to quantitative assessment of regional perfusion [45, 64]. Quantitative assessment of MBF offers clinical values over qualitative assessment of MBF, including the potential to detect balanced ischemia and absolute measurement of perfusion, which is better to grade the severity of ischemia [65].

The feasibility of dynamic CTP for the detection of hemodynamically significant coronary stenosis was investigated by Bamberg et al. [66] with invasive fractional flow reserve (FFR) as a reference standard. In the study, an MBF cut point of 75 mL/100 mL/min was determined for the differentiation between hemodynamically significant and nonsignificant coronary stenoses on the basis of maximization of the area under the curve. With use of estimated MBF to reclassify the lesions, the PPV was significantly increased from 49% to 78% compared with CCTA. The results suggest that dynamic CT-based stress MPI provide incremental diagnostic value for the detection of hemodynamically significant coronary artery stenosis. A recent study of stress dynamic CTP for the evaluation of myocardial ischemia and infarction was performed with CMR as the reference standard [67]. In this study, a threshold of 88 mL/100 mL/min for MBF was determined to indicate a perfusion defect. The diagnostic accuracy of CTP for the detection of any perfusion defect was good with 77.8% sensitivity, 75.41% specificity, 91.3% NPV, and 50.6% PPV. Higher diagnostic accuracy was defined for transmural perfusion defects with 87.8% sensitivity and infarcted segments with 85.3% sensitivity. MBV was significantly lower in infarcted segments compared with ischemic segments. The study indicates that dynamic stress CTP provides good diagnostic accuracy for the detection of myocardial perfusion defects and has the potential to differentiate ischemic from infracted myocardium. Wang et al. [63] studied the adenosine-stress dynamic CTP compared with conventional coronary angiography and SPECT. In this study, moderate correlation was observed between adenosine-stress CTP and SPECT-MPI (*r* = 0.639). The combination of CTP with CCTA improves the diagnostic accuracy for identifying flow-limiting stenosis compared with CCTA alone.

The comparison of dynamic with single-phase CT acquisition for MPI has been recently studied. One study demonstrated similar performance of single-phase peak enhancement to the perfusion parameter MBF in the detection of ischemic myocardium [68]. Another study showed that dynamic acquisition techniques allow the identification of more subtle perfusion changes at moderate stenosis (50%), whereas both techniques permit the identification of high-grade stenosis (75%). The results also suggest that dynamic myocardial CTP may be more sensitive in the detection of subtle differences of myocardial perfusion compared with single-phase perfusion imaging [69]. Currently, wide detector CT system allows for covering the entire left ventricular myocardium without table movement and is more suitable for dynamic CTP imaging [62]. Manual postprocessing of dynamic myocardial perfusion CT data has been time-consuming and laborious. Accordingly, the automated software for perfusion analyses is desirable to facilitate rapid interpretation and enhance reproducibility. Ebersberger et al. [70] reported the use of 3D semiquantitative software for the analysis of myocardial perfusion CT data with SPECT as the reference standard. The results indicate that the 3D semiquantitative software substantially decreases postprocessing and interpretation times while maintaining the diagnostic accuracy. Since dynamic myocardial CTP involves multiphase scans, dose issue is the major concern. Therefore, care must be taken to minimize the radiation dose while maintaining the clinically relevant information. The studies have shown promising results, and the use of dynamic myocardial CTP may allow for accurate assessment of the hemodynamically significant coronary stenosis. However, further research will be required to verify the clinical effectiveness of this approach.

## 6. Delayed Enhancement CT

Delayed enhancement CT was found to be feasible for the assessment myocardial infarction and viability. Hyperenhanced myocardial areas on delayed CT images may represent scar tissue caused by myocardial infarction. However, the contrast-to-noise ratio between the infarcted region and the normal myocardium is limited compared with CMR delayed enhancement imaging [52, 71]. One study suggested that delayed enhancement CT does not add incremental diagnostic value to CTP. Thus, delayed enhancement acquisition can be omitted to reduce radiation dose [42]. Current data are insufficient to support the use of delayed enhancement CT for the assessment of myocardial viability in patients with suspected CAD.

## 7. Limitations and Prospect of CT Perfusion

The main limitations of CTP currently are artifacts, radiation exposure, and contrast load. Artifacts can alter myocardial attenuation and interfere with myocardial perfusion analysis, which may result in misinterpretation of myocardial blood flow and perfusion defects [72]. It has been shown that in a group of asymptomatic patients with no history of CAD, who undergo CT perfusion angiography, artifacts in the posterobasal wall were identified in more than two-thirds of patients [73]. These low-attenuation pseudoperfusion defects can largely be ascribed to beam-hardening artifacts most likely from the adjacent bone structures. The high iodine concentration in the descending aorta and left ventricular chamber, however, can also result in beam-hardening artifacts that can mimic the appearance of myocardial perfusion defects and should be taken into account in the judgment of CT perfusion images. Therefore, beam-hardening artifact correction for coronary CT imaging is essential for accurate assessment of myocardial perfusion imaging. Recent study shows that image-based beam-hardening correction algorithm is feasible for the correction of beam-hardening artifacts that mimic perfusion defects. The beam-hardening correction used in the study was an image-based correction algorithm that enables individual estimation of the beam hardening from high-enhancing material and water-enhancing material for accurate calculation of the amount of beam hardening and to reconstruct a corrected image [74, 75]. The study also demonstrates that virtual monochromatic images generated using fast-switching dual-kVp technology are feasible for the correction of beam-hardening artifacts in myocardial CT imaging [71]. However, beam-hardening artifacts cannot be completely eliminated using the current beam-hardening correction algorithms [74]. In the near future, more rigorous correction algorithms for CTP imaging may improve accurate assessment of myocardial perfusion.

The increasing radiation exposure to the total population due to CT scans has raised serious concerns [76]. The main concern of exposure to ionizing radiation is the potential risk of cancer [77]. Another important limitation in myocardial CTP is the relatively high radiation dose. Stress testing requires repeated scanning that is associated with additional radiation exposure. In comparison with static CTP, dynamic myocardial CTP techniques involve much higher radiation exposure to the patients, because of the use of multiple phases. Notably, radiation doses are steadily decreasing as more dose-reduction strategies such as prospective ECG-gating, low tube voltage, and automatic tube current modulation are performed [78–80]. In Blankstein's study [46], the average radiation exposure for the complete CT protocol including rest perfusion, stress perfusion, and delayed enhancement scan was 12.7 ± 4.0 mSv which is similar to SPECT (12.7 ± 0.4 mSv). In a recent study, by Feuchtner et al. [33], lower radiation doses can be achieved by using the latest dual-source CT with high-pitch mode, with a mean effective dose of 0.93 ± 0.18 mSv (range: 0.75–1.48 mSv) for stress and 1.59 ± 1.3 mSv (range: 0.53–5.8 mSv) for rest imaging, respectively. The use of iterative reconstruction algorithm can improve signal-to-noise ratio (SNR) and contrast-to-noise ratio (CNR) and may facilitate radiation dose savings in CTP without influencing diagnostic quality [81].

Besides the radiation, concerns about iodine contrast associated with CTP remain to be addressed, especially in patients with abnormal renal function. Combined CCTA/CTP requires doubling the iodinated contrast dose compared with CCTA alone. The repeated scanning in stress testing requires additional iodine contrast. Recent studies have demonstrated 74% reduction in radiation and 28% reduction in contrast dose without significantly compromising the ability to detect stress-induced myocardial ischemia by using lower tube voltage [78]. Therefore, optimizing the scan protocol to reduce radiation exposure and contrast dose is important for myocardial perfusion imaging. Another limitation during performing CTP is the use of beta-blockers for heart rate control which may mask ischemia [82].

Myocardial CTP has the potential to noninvasively assess both coronary artery stenosis and its functional significance at a single examination. Current evidence indicates that the combination of CCTA with CTP imaging may improve diagnostic accuracy compared with CCTA alone for the evaluation of suspected CAD. Future advances of CT technology with improved spatial and temporal resolution and detector widths, as well as beam-hardening correction algorithms, will enhance the diagnostic accuracy of CTP. CTP would be performed with very low radiation dose and free of beam-hardening artifacts. More studies will be required to further define the diagnostic value of combined CCTA and CTP. Longer term follow-up studies are needed to validate the prognostic value of CTP, as well as the patient outcomes of CTP-guided clinical decision making. Although CTP has shown the potential to evaluate the hemodynamic significance of stenotic lesions, there is still no strong evidence to support the routine clinical use of this novel technique.

## 8. Noninvasive FFR_**CT**_

FFR is widely accepted as the gold standard invasive physiologic test for the assessment of lesion-specific ischemia [83, 84]. FFR has been shown to be valuable for the identification of lesions which can benefit from revascularization or can be safely deferred. FFR-guided percutaneous coronary intervention (PCI) results in a significant reduction in major adverse events as compared to PCI-guided by angiography alone [83]. FFR is defined as the ratio of the mean coronary pressure distal to a coronary stenosis to the mean aortic pressure during maximal coronary blood flow. It can be easily measured during coronary angiography with a coronary pressure guidewire. A FFR value of 0.80 or less identifies hemodynamic significance of coronary stenosis [85]. In comparison to MPI that identifies territory-specific ischemia, the advantage of FFR is the assessment of ischemia at the lesion level. However, FFR is an invasive procedure and is not suitable for routine patient evaluation.

Recently, The FFR can be computed from standard CCTA scans (FFR_CT_) with the advances in computational fluid dynamics and image-based modeling. FFR_CT_ is a novel noninvasive technique that can be used to determine the physiologic significance of coronary stenosis without any modification of CCTA protocols, additional image acquisition, or administration of medications [86]. FFR_CT_ is calculated by computational fluid dynamic (CFD) modeling after semiautomated segmentation of coronary tree and left ventricular mass. Three-dimensional blood flow and pressure of the coronary arteries can be simulated, with blood modeled as a Newtonian fluid with incompressible Navier-Stokes equations and solved subject to appropriate initial and boundary conditions with a finite element method on parallel supercomputer [87, 88]. The FFR_CT_ is modeled for conditions of adenosine-induced hyperemia without adenosine infusion. This process is computationally complex and time-consuming and may require several hours. Similar to invasive FFR, FFR_CT_ was obtained by dividing the mean pressure distal to the coronary stenosis by the mean aortic pressure. An FFR_CT_ < 0.80 was considered diagnostic of lesion-specific ischemia [86]. Up to now, limited FFR_CT_ studies have been performed. In the DISCOVER-FLOW (Diagnosis of Ischemia-Causing Stenoses Obtained Via Noninvasive Fractional Flow Reserve) trial [86], FFRCT was performed on 159 vessels in 103 patients with suspected or known CAD undergoing CCTA and invasive coronary angiography. The diagnostic performance of FFR_CT_ and CCTA stenosis for the detection of ischemia-causing lesions was assessed with invasive FFR as the reference standard. The results demonstrate that the per-vessel accuracy, sensitivity, specificity, PPV, and NPV of FFR_CT_ were 84.3%, 87.9%, 82.2%, 73.9%, 92.2%, respectively, and were 58.5%, 91.4%, 39.6%, 46.5%, 88.9%, respectively, for CCTA stenosis. The area under the ROC was 0.90 for FFR_CT_ and 0.75 for CCTA (*P* < 0.001). The specificity and PPV were remarkably improved by FFR_CT_ compared with CCTA stenosis. The DeFACTO (Determination of Fractional Flow Reserve by Anatomic Computed Tomographic Angiography) trial [89], a multicenter international study which consisted of 252 patients, was designed to evaluate the performance of noninvasive FFR_CT_ for the diagnosis of ischemia with invasive FFR as a reference standard. On a per-patient basis, diagnostic accuracy, sensitivity, specificity, PPV, and NPV of FFR_CT_ plus CCTA were 73%, 90%, 54%, 67%, and 84%, respectively. When comparing FFR_CT_ with CCTA stenosis for diagnosing obstructive CAD, FFR_CT_ demonstrated improved discrimination with area under the ROC 0.81 for FFR_CT_ and 0.68 for CCTA (*P* < 0.001). The study results demonstrated improved diagnostic accuracy of FFR_CT_ plus CCTA versus CCTA alone for the diagnosis of ischemia, although the study did not achieve its prespecified primary outcome goal of diagnostic accuracy of greater than 70% of the lower bound of the sided 95% confidence interval (95% CI, 67%–78%). The study suggests the potential of FFR_CT_ as a promising noninvasive method for the identification of individuals with ischemia. More recently, the multicenter study NXT (Analysis of Coronary Blood Flow Using CT Angiography: Next Steps) trial [90] included 251 patients scheduled to undergo clinically indicated invasive coronary angiography for suspected CAD. The diagnostic performance of FFR_CT_ for the diagnosis of ischemia was determined with invasive FFR as the reference standard. As compared to previous studies [86, 89], the improved FFR_CT_ technology was used with an emphasis on CCTA image quality. The area under the ROC curve for FFR_CT_ was 0.90 versus 0.81 for CCTA (*P* = 0.0008). On per-patient basis, sensitivity and specificity to identify ischemia were 86% and 79% for FFR_CT_ versus 94% and 34% for CCTA, respectively. When compared to CCTA, the improved diagnostic performance of FFR_CT_ is in particular with regard to specificity. The study demonstrates that FFR_CT_ has relatively high diagnostic accuracy compared to invasive FFR for identifying hemodynamically significant CAD.

FFR_CT_ technology enables the “virtual stenting” modeling and may be served to predict hemodynamic effect of coronary stenting on ischemia-causing stenoses [91]. Kim et al. [92] studied the application of FFR_CT_ in 44 patients (48 lesions) to predict FFR value changes after stenting with invasive FFR as the reference standard. Before intervention, invasive FFR was 0.70 ± 0.14 and increased to 0.90 ± 0.05 after stenting. FFR_CT_ was 0.70 ± 0.15 before intervention and increased to 0.88 ± 0.05 after virtual coronary stenting. There was a good correlation between FFR and FFR_CT_ before (*R* = 0.60; *P* < 0.001) and after stenting (*R* = 0.55; *P* < 0.001). This study demonstrates that virtual stenting by FFR_CT_ is feasible and may be helpful for revascularization planning before invasive procedures.

## 9. Limitations and Prospect of FFR_**CT**_

There are several limitations for physiologic assessment of coronary stenosis by FFR_CT_. First, FFR_CT_ is calculated by computational simulation of adenosine mediated hyperemia rather than by actual administration of adenosine. Second, the value of FFR_CT_ is influenced not only by stenosis severity but also by the presence of viable or scarred myocardium [93]. Third, the calculated FFR_CT_ values may be lower than those of measured FFR in patients with microvascular disease, for the modeling of adenosine-induced hyperemia may overestimate the degree of vasodilation [88]. Fourth, because FFR_CT_ requires accurate anatomic models, numerous artifacts on CCTA may affect FFR_CT_ calculation, including the presence of heavy calcification, motion artifacts, lower SNR, and misregistration. Therefore, CCTA data with good image quality is essential for the accuracy of FFRCT interpretation.

The results of the limited studies indicate that FFR_CT_ is feasible for noninvasive determination of lesion-specific ischemia. Although the accuracy of FFR_CT_ was reported as modest in several multicenter trials, this novel technique shows promise not only for diagnosing lesion-specific ischemia but also for predicting revascularization benefit. The present studies support the potential of CCTA with FFR_CT_ as a gatekeeper to invasive coronary angiography and revascularization. Further improvements in the technology or new algorithms for a reliable estimation of FFR will be a prerequisite for wide acceptance of FFR_CT_, and further studies are needed to determine its clinical utility compared with other noninvasive testing. Combination of CCTA and FFR_CT_ might permit evaluation of CAD in a “one-stop-shop” approach in the future.

## 10. Transluminal Attenuation Gradient (TAG)

Assessment of the change in intraluminal contrast attenuation across a coronary stenosis on CCTA may allow prediction of functional significance of coronary stenosis [94]. A methodology called “coronary opacification” (CO) difference, defined as the contrast attenuation difference across a stenosis, has been proposed for functional prediction of stenosis. In the study, by Chow et al. [95], attenuation values of coronary lumen were measured before and after stenoses and normalized to the descending aorta on the same axial slice, because the image acquisition is not temporally uniform using 64-slice CT. Corrected coronary opacification (CCO) differences were compared with the severity of coronary stenosis and thrombolysis in myocardial infarction (TIMI) flow at invasive coronary angiography. The results indicate that changes in CCO across coronary stenoses seem to predict abnormal resting coronary blood flow.

However, the measurement of gradients across coronary stenoses is inherently more robust than evaluation of opacification difference. Transluminal attenuation gradient (TAG) is defined as the linear regression coefficient between luminal attenuation (Hu) and axial distance along the vessel from the coronary ostium. TAG is reflected by the kinetics of the iodinated contrast media within the coronary arteries. Study confirmed that opacification gradients exist in patients with normal coronary arteries. The TAG along the course of a coronary artery can be reproducibly evaluated with CCTA [96]. The hypothesis of TAG as a functional test is that contrast attenuation should fall off more rapidly across a lesion with functionally significant stenosis [97]. Recent studies suggest that TAG may provide useful information for the assessment of functional significance of coronary stenosis with the comparison of invasive FFR. TAG significantly improves both sensitivity and specificity over CCTA stenosis degree alone [94].

It has been reported that TAG has the potential to improve the classification of stenosis severity on 64-slice CCTA compared with coronary angiography as a reference standard, especially in calcified lesions, which is particularly useful because CCTA is limited in accuracy when lesions are calcified [98]. The limitation of TAG analysis, using 64-slice CCTA, pointed out by the authors, is that attenuation along a vessel does not reflect contrast density at a single time point, potentially limiting the diagnostic performance of the TAG method. The use of 320-detector row CT with whole-heart volume to be imaged in a single gantry rotation enables noninvasive quantitative assessment of coronary contrast changes with temporal uniformity, which may be ideal for TAG functional assessment of a coronary arterial stenosis [94, 96]. Comparing labor-intensive manual TAG calculation methods, latest automated gradient software package can reduce computation time with high accuracy and reproducibility [99].

Combined TAG and CCTA assessment may have incremental value over CCTA alone for detecting functionally significant coronary stenoses, especially in severely calcified lesions. TAG depends on the luminal attenuation values, and the accuracy of quantification may be influenced by various artifacts on CCTA. So, maintaining image quality of CCTA is important for TAG analysis. TAG assessment has particular attraction, as it does not require additional scan or complex computation compared with CT perfusion or FFR_CT_. However, given the limited evidence so far, larger studies with current acquisition and reconstruction protocols as well as new analysis software are required to validate the diagnostic and prognostic value of this approach.

## 11. Summary

CCTA has become an important noninvasive imaging modality in the diagnosis of CAD. CCTA enables accurate evaluation of coronary artery stenosis. However, CCTA provides limited information on the physiological significance of stenotic lesions. There is a great interest in evaluating both coronary anatomy and its hemodynamic significance during a single examination. Combination of anatomic and physiologic information would be beneficial for clinical decision making, particularly in lesions with moderate stenoses. Recently, novel CT techniques which can provide both anatomical and functional assessments of CAD were developed, including myocardial CTP and noninvasive FFR_CT_, which simulate FFR from CT data using CFD method and TAG derived from the gradient in contrast opacification along a coronary artery. The current studies have demonstrated that these methods are feasible for noninvasive assessment of CAD and have the potential to provide incremental value in detecting functionally significant coronary stenosis over CCTA alone. Although the currently available data are promising, these approaches are still in its early stage, and their diagnostic values still need further validation. Further research is required to identify the prognostic value and clinical outcomes of decision making based on these techniques.
